# Beyond the Hospital Residential Aged Care Home Referral: A Critical Realist Exploration of Residential Aged Care Admission Practices Directly From Hospital

**DOI:** 10.1111/ajag.70203

**Published:** 2026-07-11

**Authors:** Angel Carrasco, Rachna Kumar, Natalia Mendez Arostica, Gunjan Patil, Hendrika Laetitia Hattingh

**Affiliations:** ^1^ Social Work Services Gold Coast Hospital and Health Service Southport Queensland Australia; ^2^ School of Allied Health, Sport and Social Work Griffith University Southport Queensland Australia; ^3^ Allied Health Research Gold Coast Hospital and Health Service Southport Queensland Australia; ^4^ School of Pharmacy and Medical Sciences Griffith University Southport Queensland Australia; ^5^ School of Pharmacy and Pharmaceutical Sciences The University of Queensland Brisbane Queensland Australia

**Keywords:** discharge planning, nursing home, patient handoff, residential aged care facility

## Abstract

**Objective:**

This study explored the complex decision‐making processes underpinning hospital‐to‐permanent Residential Aged Care Home (RACH) transitions from the perspective of RACH managers.

**Methods:**

Using a critical realist methodology, semi‐structured interviews were conducted with 15 RACH managers on the Gold Coast, Queensland, Australia. Data were analysed inductively using reflexive thematic analysis to identify patterns and underlying causal mechanisms that shape admission practices.

**Results:**

Five interrelated themes emerged: (1) structural and organisational constraints; (2) screening as organisational risk; (3) resident–facility compatibility; (4) relational and legal negotiation; and (5) logistical coordination. Findings revealed that hospital‐to‐RACH admission decisions are influenced not only by observable procedural elements but also by deeper systemic mechanisms, including funding structures, staffing limitations, resident mix and policy constraints, which contribute to prolonged hospital wait times for patients awaiting permanent placement.

**Conclusions:**

The study presents a conceptual model of hospital‐to‐RACH placement, highlighting the operational realities faced by RACH managers. Findings underscore the need for integrated discharge planning, flexible funding arrangements, and systemic support such as workforce training and cross‐sector collaboration to improve the efficiency and quality of care transitions.

## Policy Impact

Policy reform is needed to better align hospital discharge imperatives with the operational capacities of Residential Aged Care Homes, ensuring timely and equitable access to permanent residential care.

## 1 Introduction

The rising number of older people remaining in public hospitals while awaiting permanent residential aged care placement represents a critical and escalating system‐level failure [1, 2]. Across Australia, older patients who are medically stable continue to occupy acute hospital beds for extended periods because appropriate Residential Aged Care Home (RACH) placements are unavailable or delayed. These prolonged hospital stays are associated with functional decline, increased risk of hospital‐acquired complications, distress for patients and families, and sustained pressure on already stretched acute care hospitals.

Timely and appropriate admission from hospital to RACH is a critical aspect of safe discharge planning for hospitalised older adults. Although structured referral systems and aged care legislation are in place, health systems continue to face significant challenges related to prolonged discharge waiting times and pressure on acute hospital beds while older patients await RACH placements, including inappropriate RACH placements leading to readmissions of older patients [2]. As Australia's population ages and the prevalence of complex, chronic health conditions increase, the demand for residential aged care is expected to significantly escalate and continue. This issue places increasing pressure on both public hospitals and the RACH sector to provide high‐quality, coordinated services that address the multifaceted needs of older people, particularly those waiting for RACH placement in public hospitals [2, 3].

National data indicate that hospitalisation remains a significant pathway into residential aged care in Australia. In 2023–2024, approximately 75,600 people entered permanent residential aged care as part of nearly 299,000 total aged care admissions [3]. Linked analyses of people living in Australia aged 65 years and over show that around 23% entered residential aged care within 7 days of hospital discharge, increasing to over one‐third within 12 months, with substantially higher rates among people living with dementia [4, 5]. These findings suggest that hospitalisation often functions as a major point of entry into long‐term RACH placement. Individuals transitioning from hospital to RACH commonly present with multimorbidity, cognitive impairment and functional decline, reflecting high levels of clinical and support needs. Ongoing hospital system pressures, including delays in discharge and an increasing number of long‐stay patients due to limited aged care availability, further underscore the importance of coordinated discharge planning and integration between acute hospitals and the RACHs to ensure safe and timely transitions [3].

Australian RACH funding operates through a hybrid public‐private model, combining Commonwealth government subsidies under the Australian National Aged Care Classification (AN_ACC) framework based on level of care required by residents alongside regulated resident contributions (including basic daily fees, means‐tested care fees and accommodation payments) [6]. The Australian RACH funding model reflects efforts to balance care needs‐based government funding with fiscal sustainability of the RACH sector through private means‐tested resident contributions [7, 8]. Residents who are unable to contribute towards these accommodation costs and contributions due to limited financial means receive ‘concessional’ funding from the government providing a subsidised financial supplement to the RACH on their behalf. Within this concessional funding model, this supplement is typically lower than revenue generated through resident‐paid accommodation contributions which may influence provider capacity and willingness to accept residents. Research highlights that workforce shortages, equity disparities and funding inadequacies remain key structural challenges, affecting quality and sustainability despite policy reforms [7, 9, 10, 11]. Regulation by the Aged Care Quality and Safety Commission links funding to compliance with quality standards, though enforcement is only weakly tied to measurable outcomes [12]. Overall, although reforms have aimed to improve needs‐based financing and staffing capacity, systemic, financial and operational complexities continue to challenge long‐term sustainability, equity and quality in the sector [13, 14, 15].

Residential Aged Care Home managers play a pivotal role in the timely acceptance of hospital‐referred patients awaiting permanent placement. Their decisions are central to facilitating safe and effective transitions of care and mitigating the risks associated with extended hospital stays in older adults, including hospital‐acquired infections, functional decline, falls, malnutrition and delirium. Despite the significance of their role, the processes and considerations informing these decisions remain poorly understood.

Although discharge planning to a permanent RACH placement may seem straightforward in principle, in practice, it is often complicated by logistical challenges, differing perspectives and processes between hospital and aged care settings, often hindering smooth care and timely transitions [16, 17]. Communication breakdowns between healthcare providers are well‐documented and have been shown to compromise care quality and patient safety. Delays in discharge decision‐making, prolonged hospital stays, and the phenomenon of ‘bed‐blocking’, where patients remain hospitalised despite being medically ready for discharge due to the unavailability of appropriate aged care placements, are common occurrences. These issues have been reported internationally, including in Austria, the United Kingdom, and Australia, contributing to broader systemic concerns around workforce shortages, hospital capacity, and resource allocation [9, 18, 19].

Efforts to improve hospital‐to‐RACH transitions have centred on enhancing the quality and timeliness of information exchange at discharge. Strategies have included the revision of referral processes [20], implementation of quality improvement tools such as process mapping [21], and the introduction of ‘warm handoffs’ between hospital and aged care providers to ensure relational continuity [22]. Although these approaches differ in execution, they share the common aim of fostering collaboration across the care continuum to reduce discharge‐related risks and improve outcomes.

In Australia, empirical research examining the implementation and effectiveness of hospital‐to‐residential aged care discharge strategies from the RACH managers' perspective remains relatively limited. A 2022 study of two public hospitals in Brisbane, Queensland, identified promising discharge planning practices; however, the findings were context‐specific and embedded within particular teams and patient populations, limiting broader generalisability [23]. This underscores the need for a more comprehensive and nuanced understanding of the discharge process, particularly from the perspective of RACH managers who are directly responsible for assessing referrals and determining admission suitability.

This study addresses this gap by examining hospital‐to‐RACH admissions through the lens of RACH managers' decision‐making. Guided by critical realist theory, it moves beyond descriptive accounts of discharge delay to interrogate the structural, organisational, and relational mechanisms that shape admission outcomes. In doing so, it explores how sector‐wide funding arrangements, regulatory expectations, workforce constraints, and hospital discharge pressures identified in the literature as systemic issues are interpreted and enacted in everyday managerial practice. By illuminating how these underlying systemic mechanisms influence day‐to‐day decision‐making, the study offers a deeper explanatory account of prolonged hospital stays from the RACH managers' perspective and the mechanisms that sustain them.

## 2 Methods

This study employed an interpretive qualitative design informed by critical realist theory to examine how RACH managers' perceptions and experiences shape hospital‐to‐RACH admission decisions. Critical Realism posits that social outcomes are generated by underlying structural and organisational mechanisms that exist independently of individuals but are interpreted and enacted through human reasoning and agency [24, 25]. Within this framework, admission decisions are understood as observable outcomes produced through the interaction of sector‐level structures, organisational constraints and managerial judgement.

Residential Aged Care Home managers' accounts were therefore treated as situated interpretations of real conditions, including funding arrangements, regulatory requirements, workforce capacity and hospital discharge pressures [26, 27]. Adopting a critical realist methodology enabled the study to examine not only what occurs in hospital‐to‐RACH transfers, but why contested, delayed or declined transitions persist despite ongoing policy reform. Particular attention was given to identifying how structural pressures are absorbed, negotiated and operationalised at the hospital–aged care interface.

Analysis was guided by a context–mechanism–outcome (CMO) framework to explore how structural conditions (context) activated organisational and relational mechanisms that shaped admission decisions (outcomes) across different facility settings. To support this analysis, findings were examined across macro (system), meso (organisational) and micro (individual) levels, consistent with a critical realist approach to understanding how mechanisms operate across layered social contexts [28]. This approach facilitated the development of an explanatory account capable of identifying leverage points for improved system alignment and more sustainable discharge practices.

### 2.1 Participants and Setting

This study was undertaken on the Gold Coast region of Australia with a population of approximately 743,000, of which 111,000 residents were aged 65 years and over, a demographic proportion that is increasing rapidly and exceeds state and national averages [29, 30]. Two main public hospitals—Gold Coast University Hospital and Robina Hospital—were identified as the main hospital facilities of Gold Coast Hospital and Health Service (GCHHS) which interfaced with the region's 55 RACHs [31].

Residential Aged Care Homes were recruited purposively, consistent with a critical realist approach, with the research team aiming to capture diversity across the 54 RACHs identified on the Gold Coast. The sample included a range of facility sizes, both for‐profit and not‐for‐profit, with only one facility approached from organisations operating multiple sites. Initial contact was made with managers directly responsible for hospital‐to‐RACH admission decisions, who were invited to participate. Recruitment followed a rolling approach until sufficient information power was achieved, ensuring data richness to explore organisational and system‐level mechanisms [32]. All 15 managers approached agreed to participate, identified via the Queensland Hospital Admitted Patient Data Collection. Participants were included if they held decision‐making responsibility for admissions; staff without authority, managers outside the Gold Coast, hospital clinicians, family members and policymakers were excluded. This approach maintained focus on how RACH managers interpreted and enacted structural and organisational mechanisms in practice, consistent with the critical realist aim of examining the generative mechanisms shaping hospital‐to‐RACH admissions [24, 26].

### 2.2 Data Collection

Semi‐structured interviews were conducted face‐to‐face or via Microsoft Teams by hospital‐based social workers with prior professional experience referring patients to RACHs. The interviewers' familiarity with RACH managers was treated as part of the research context, helping to generate rich, detailed insights while reflexive practices, which included the research team meeting regularly with the interviewers, ensured that interpretations accurately reflected participants' perspectives and supported identification of the mechanisms shaping hospital‐to‐RACH admission outcomes [26, 27]. The interview questions were collaboratively developed and informed by a critical realist orientation [24]. They focused on admission processes, structural requirements, and the barriers and enablers influencing RACF acceptance of hospital referrals, moving beyond subjective accounts to examine organisational and regulatory mechanisms shaping practice, including comparison between current and ideal processes to explore underlying system‐level influences (see Supporting Information). Interviewers used open‐ended questions with conversational prompts exploring referral assessment, organisational and clinical constraints, behavioural management, staffing considerations and logistical coordination, encouraging participants to share experiences while enabling probing of operational and structural mechanisms influencing decisions. Interviews were audio‐recorded with participant consent, and transcribed verbatim.

### 2.3 Sample Size and Rationale

A target sample size of 15 participants was determined using principles of information power, emphasising the depth and relevance of data over numerical adequacy [32]. Given the specific focus on hospital‐to‐RACH transitions, the high level of participant engagement, expertise, and the aim to capture context‐dependent mechanisms, this sample was sufficient to develop a robust explanatory account of decision‐making processes.

### 2.4 Data Analysis

Data were analysed inductively and thematically using reflexive thematic analysis [33]. Two researchers conducted initial coding and theme development, with a third researcher reviewing coding to ensure analytical rigour. NVivo software supported data organisation and coding.

The research team met regularly to discuss participant feedback, refine coding frameworks and interrogate emerging interpretations. These discussions employed retroductive reasoning, moving beyond descriptive accounts towards theorising the causal mechanisms and contextual configurations most likely to explain observed transfer outcomes [27, 33]. Data collection continued until explanatory saturation was reached, defined as the point at which additional interviews did not substantively add new insights into mechanisms shaping hospital‐to‐RACH transfers. This was reached at 15 participants, and this decision was supported with discussion with the research team. In line with critical realist principles, the aim was not exhaustive thematic saturation but to develop a robust explanatory account of how and why transfer outcomes occurred across varied organisational contexts [26, 27].

## 3 Results

Fifteen interviews were conducted with RACH managers September–November 2024, with interviews lasting 35–90 min.

### 3.1 RACH Manger and RACH Facility Characteristics September–November 2024

Table 1 indicates that most participants held primarily administrative roles (60%), with one‐third identifying as registered nurses (33%) and a small proportion (7%) combining clinical and administrative responsibilities. Experience levels were varied, with 40% reporting 3 years or less in management roles and one‐third reporting seven or more years. This spread reflected a mix of emerging and experienced leaders navigating contemporary aged care reforms and system pressures. Most participants held vocational or tertiary qualifications (80%), indicating a relatively skilled managerial workforce. Facility representation was concentrated in for‐profit services (67%) and medium‐to‐large facilities (93%), with over half working in large homes (> 101 beds).

**TABLE 1:** Summary of RACH manager and facility characteristics.

Characteristic	Category	*n*	%
Role	Clinical (RN)	5	33
	Administrative	9	60
	Both clinical & administrative	1	7
Practice experience (years)	0–1	3	20
	1–3	3	20
	4–6	4	27
	7–9	2	13
	10+	3	20
Education	Secondary	3	20
	Diploma	6	40
	Bachelor's (completed/in progress)	6	40
Facility type	For‐profit	10	67
	Not‐for‐profit	5	33
Facility size category	Small (< 60 beds)	1	7
	Medium (61–100 beds)	6	40
	Large (> 101 beds)	8	53

Abbreviations: RACH, Residential Aged Care Home; RN, registered nurse.

### 3.2 Thematic Analysis of Interviews

**FIGURE 1:**
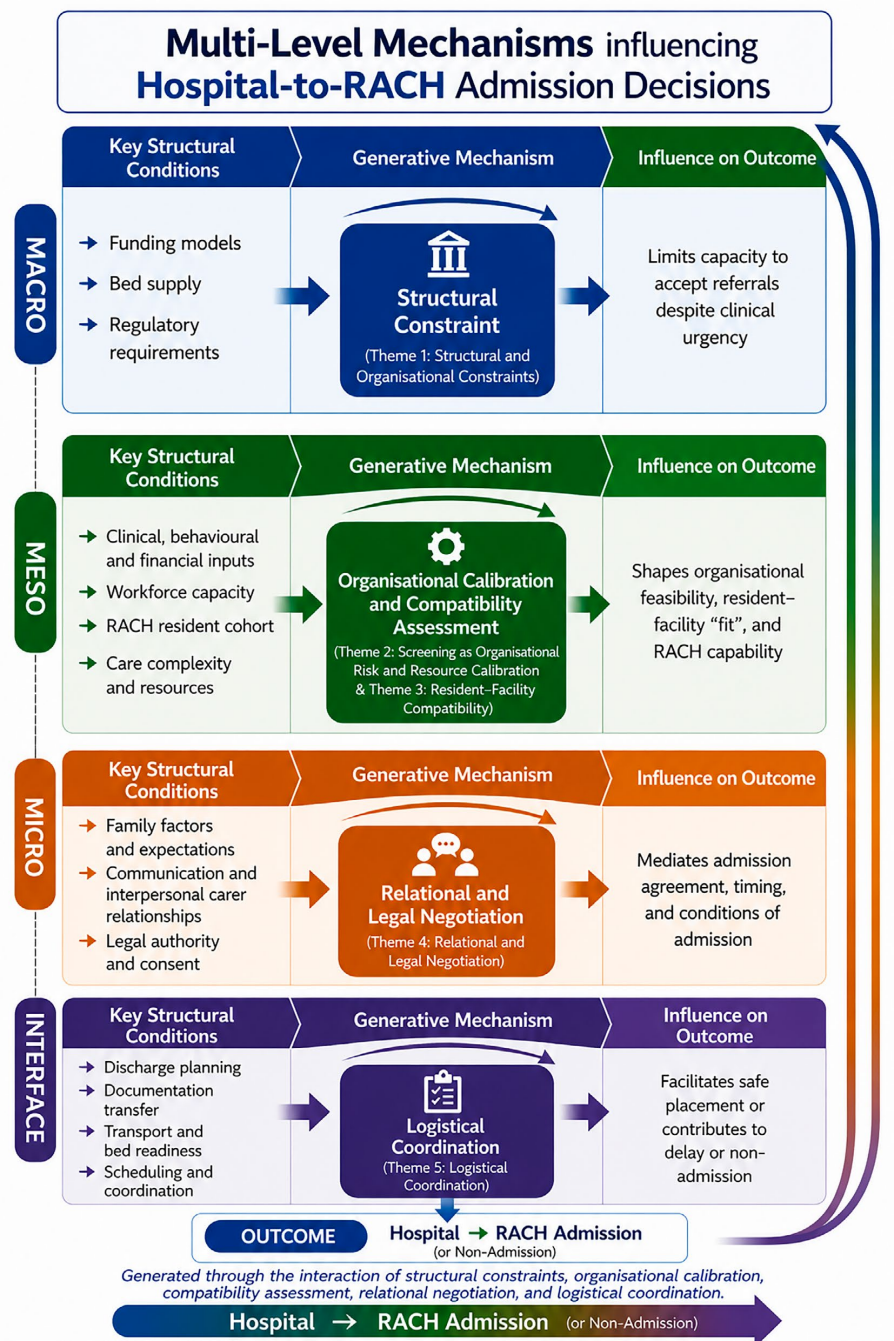
Multi‐level mechanisms influencing hospital‐to‐RACH admission decisions.

Data analysis identified five interrelated themes shared across the RACH managers, illustrating the key processes and decision‐making factors and processes involved in admitting patients directly from hospital to RACHs. These themes captured both the observable practices described by participants and the underlying structural mechanisms influencing their decisions, aligning with the principles of a critical realist framework. A summary of these themes and associated decision‐making processes is presented in Figure 1.

This model explains hospital‐to‐RACH admission decisions as the product of interacting mechanisms operating across macro, meso, micro and interface levels. Although five themes were identified in this study, these operated across four system levels as indicated in the above model. Notably, screening as organisational risk and resource calibration, and resident–facility compatibility both functioned as meso‐level organisational mechanisms and were therefore represented together within the model. The model highlights how structural conditions constrain managerial discretion, organisational risk calibration determined resident–facility fit, relational negotiation shaped agreement, and logistical coordination enabled (or delayed) safe transfer. Admission or non‐admission therefore emerged from the combined effects of systemic, organisational, relational and operational processes rather than from discharge processes alone.

### 3.3 Structural and Organisational Constraints Conditioning Referral Decisions

Although referrals followed a formal, standardised process, managers' ability to accept patients was often constrained by macro‐level structural mechanisms operating at the policy sector and facility levels. Decisions about hospital referrals were frequently shaped by bed availability, funding models, organisational policy, and regulatory governance, often preceding hospital clinical urgency.

Limited bed supply emerged as a key constraint:

The biggest barriers we have are no beds like you. We have no beds! (P2)

Funding classifications—particularly concessional status—interacted with bed scarcity to limit admission options:

So, I look at that and see concessional and think, oh no, I don't even download anything. I don't even look at it really, which is unfortunate. (P11)

Broader medication regulatory and governance structures also shaped decision‐making by constraining the capacity of RACHs to manage residents requiring psychotropic medications or restrictive practices:

We are very heavily governed around that (psychotropic medications). We have a lot of red tape to be able to utilise those things, whereas I think the hospitals can use those a bit easier than we can. But when they come to us, we can't, we can't give them those psychotropics … So that's when we go ‘we can't handle them’. (P14)

Within this model, these factors functioned as generative mechanisms operating within broader structural conditions that limited managerial decision‐making and influenced whether hospital transfers were accepted. Although hospital referrals were often clinically urgent, RACH managers were frequently unable to prioritise them due to funding arrangements, regulatory requirements, and local workforce or service capacity constraints. As a result, transfer decisions were shaped not only by patient acuity, but by the interaction between organisational, policy and resource pressures.

### 3.4 Screening as Organisational Risk and Resource Calibration

At the organisational (meso) level, managers translated structural constraints into practical decision‐making screening processes. Screening acted as the key mechanism through which organisational risk and resource alignment were managed. Residential Aged Care Home managers described it as structured yet interpretive, combining clinical, behavioural, financial and legal assessments to determine whether resident needs could be safely accommodated within existing workforce, resident cohort and financial parameters.

Cognition, mobility, and overall care needs were central to this assessment:

Cognition and mobility for us is the biggest thing… it's what we need to know to work out where to place them. (P13)

Clinical requirements, such as PEG feeding, were assessed relative to staffing and unit capacity. Documentation, including ACAT support plans, NSAF codes and nursing summaries, informed this process. Behavioural risk—particularly physical aggression—was reviewed, sometimes prompting requests for Behavioural Support Plans. Financial thresholds, AN‐ACC implications, staffing availability and legal documentation (e.g., EPOA, guardianship orders) were also considered.

Within this model, screening functioned as a calibration mechanism through which macro‐level constraints are operationalised at the facility level. It functioned not simply as clinical assessment, but as an organisational process that calibrated risk, resource allocation and financial sustainability into practical admission decisions.

### 3.5 Resident–Facility Compatibility as a Stabilising Mechanism

Following screening, managers assessed whether a prospective resident was a suitable ‘fit’ for a specific bed and cohort. Compatibility decisions extended beyond clinical eligibility to consider unit stability, interpersonal dynamics and collective safety. This stage reflected a stabilising mechanism, concerned with maintaining RACH facility equilibrium, resident safety and workforce sustainability.

My memory support unit [is] predominantly females… If I add a male resident, it's going to really blow out. (P10)

Managers also balanced workload and care complexity:

If we've got a lot of high care needs residents… we want somebody that might be a little bit easier. (P11)

Behavioural risk was considered in relation to duty of care for existing residents. Compatibility was shaped by room configuration, security level, staffing skill mix and funding constraints. In this way, ‘right fit’ illustrated how organisational mechanisms stabilise systems under pressure, maintaining unit stability, managing risk and preserving care quality. Transfer outcomes were influenced by the interaction between individual needs, resident mix and operational capacity.

### 3.6 Relational and Legal Negotiation in Admission Progression

Once internal suitability was established the process shifted to the micro‐level relational domain, where admission progression depended on relational and legal domains. Here, relational mechanisms, including communication, trust‐building, expectation management and legal verification—mediated the transition from provisional acceptance to confirmed admission:

If the family are difficult with the level of referrals that are available at the moment, I'll probably move on. (P8)

We like to meet with the families from who are coming to aged care, we do tend to look at a few important documents. When I say important(document) that the client has an EPOA [Enduring Power of Attorney] in place. (P9)

This phase fulfilled legal and consent requirements while allowing mutual evaluation. Family engagement included tours, information exchange and completion of admission paperwork, alongside clarification of financial obligations. Legal instruments, such as enduring powers of attorney or guardianship arrangements, intersected with organisational protocols and relational dynamics.

Relational negotiation therefore functioned as a mediating mechanism, linking organisational decision‐making with the social realities of care transition.

### 3.7 Logistical Coordination at the Hospital–RACH Interface

The final stage of transfer was shaped by logistical coordination between hospital discharge systems and RACH workflows. Timing, documentation, transport and medication management were critical to safe transitions.

Managers liaised with hospital teams to obtain discharge summaries, medication charts and medication supplies. Admissions were often preferred earlier in the day to allow assessment and medication reconciliation. Ambulance transport was commonly used to support safe handover:

I always say to the social worker that we need that discharge summary and the medications, the seven days' worth of medication. (P1)

We prefer to take a resident in early in the week if they are behavioural, if they're not, we don't really have a problem with it. (P7)

So QAS [Queensland Ambulance Service] morning transfers are super important, but we're finding lately, QAS is under a huge amount of pressure and we're seeing that Mondays are a real issue, so sometimes we're leaving the admissions until Tuesdays because we know that Monday transfers are really hard. (P12)

Although procedures varied across facilities, coordination was influenced by staffing patterns and operational routines. Even minor misalignments could create risks affecting resident acceptance to the RACH and care continuity.

Logistical coordination therefore acted as a final stabilising mechanism at the hospital–RACH interface, ensuring a timely, safe and sustainable placement. Even when macro, meso and micro processes aligned to support admission, breakdowns at the interface could delay or prevent transfer.

## 4 Discussion

This study extends the evidence base on hospital‐to‐residential aged care home (RACH) transitions by shifting attention from hospital discharge processes to the decision‐making practices and perceptions of RACH managers. Rather than simply describing operational barriers to transfer, it applies a critical realist lens to explain how structural, organisational, unit‐level, relational and operational mechanisms interact to shape observable admission outcomes. Managers in this study navigated complex decisions within constraints, such as bed availability, funding classifications, staffing capacity, regulatory requirements, hospital discharge pressures and organisational directives.

The managerial and facility characteristics of the research participants in this study contextualise how these mechanisms were enacted. Most participants held primarily administrative leadership roles (60%), with a smaller proportion identifying as registered nurses (33%) or combining clinical and managerial responsibilities (7%). This profile indicates that admission decisions were often situated within governance, financial and operational domains rather than purely clinical frameworks, although clinical reasoning remained embedded within assessment processes. Experience levels were varied, reflecting a mix of emerging and more established leaders navigating contemporary reform and regulatory environments. These organisational contexts are important, as ownership model and scale may influence risk tolerance, resource calibration and admission thresholds. Collectively, the sample reflected admission decision‐making occurring predominantly within administratively led, market‐oriented, medium‐to‐large residential aged care settings.

Further, by examining how these macro‐ and meso‐level conditions are interpreted and enacted through everyday managerial reasoning, the study reframes prolonged hospital stays not as isolated discharge inefficiencies but as consequences of systemic misalignment between acute and aged care sectors. It makes visible the discretionary judgement work of RACH managers and demonstrates how broader policy and funding settings are translated into micro‐level admission decisions. These dynamics contribute to wider system pressures, including the growing number of older people who are medically ready for discharge but remain in public hospitals due to limited aged care placements. This is further highlighted across Australia, with more than 3000 aged care patients reportedly remaining in hospital beds, contributing to prolonged stays and ‘bed‐blocking’ [1, 2].

The five themes identified in this study provide a multi‐level explanatory model of hospital‐to‐RACH admissions. Structural and organisational constraints conditioning referral decisions highlighted how sector and facility factors—including limited bed supply and funding rules (e.g., concessional versus RAD)—limited managers' latitude to accept referrals, even in the presence of clinical urgency. Screening as organisational risk and resource calibration demonstrated how clinical, behavioural, financial and legal data are interpreted and synthesised into actionable decisions aligned with staffing and infrastructure capacity. Resident–facility compatibility reflected how managers assessed the ‘right fit’ between prospective residents and existing cohorts to maintain unit stability, group cohesion and safety. Relational and legal negotiation captured how engagement with families and legal decision‐makers mediates progression from provisional acceptance to confirmed admission, and logistical coordination emphasised how timing, documentation, transport and medication management are critical to safe transitions. Together, these themes illustrated how structural scarcity and organisational logics are enacted through interpretive work on the ground, yielding outcomes that helped explain why many medically fit older adults remain in hospital awaiting permanent care [26, 27].

These findings align with broader evidence on discharge delays and ‘bed‐blocking’ in the acute care system. Older adult patients who are medically ready for discharge but have no appropriate destination occupy valuable hospital resources, contributing to delays in emergency department access, reduced acute care capacity, and increased risk of hospital‐acquired complications, such as falls and infections [34, 35, 36]. Workforce shortages, funding inequities and regulatory constraints exacerbate these issues, particularly in regions with rapidly ageing populations [37]. Public hospitals continue to bear clinical and financial responsibility for patients awaiting RACH placement, while associated costs and risks are not proportionately transferred to RACHs, creating disincentives for facilities to admit high‐needs hospital patients.

Consistent with the multi‐level model, addressing these challenges requires layered reform. At the macro level, funding and policy settings that disincentivise admission of complex hospital patients warrant review. Incentives aligned across acute and aged care sectors, expansion of RACH capacity in high‐demand regions, workforce development and strengthened clinical leadership are essential to sustainably widen admission thresholds. At the meso level, organisational supports that enhance managers' capacity to calibrate risk—including access to behavioural support services, outreach and liaison partnerships, and clearer alignment between regulatory expectations and operational realities—may reduce risk‐averse decision‐making within existing structural limits. At the micro and interface levels, improvements in coordination can mitigate avoidable delays where structural capacity exists. Timely and comprehensive referral documentation, early identification of likely RACH candidates, integrated case conferencing, structured ‘warm handovers’, liaison roles bridging hospital and RACH systems, and standardised referral tools may reduce friction and uncertainty in transfer processes. Importantly, such strategies optimise flow but cannot fully compensate for underlying structural scarcity.

Addressing these challenges requires coordinated legislative, policy, and operational reforms, alongside practical hospital‐based strategies that can improve the flow of patients and information to RACHs [1, 18]. Expanding RACH capacity in Australia remains essential to sustainably accommodate increasing hospital demand, particularly in rapidly ageing regions. Legislative and funding reforms that incentivise admission of higher‐needs long‐stay patients waiting at hospitals, coupled with workforce development and strengthened clinical leadership, may improve hospital‐to‐RACH transitions while maintaining safe, high‐quality care.

Hospital‐to‐RACH admissions operate within a complex, multi‐layered system, where structural, operational, and relational mechanisms interact. Strategies that improve information quality, enhance coordination, and incentivise RACH acceptance of high‐needs patients are critical to reducing long‐stay hospitalisation, optimising patient outcomes and supporting timely transitions into permanent aged care. By making visible the discretionary and stabilising work of RACH managers, this study contributes a nuanced explanatory account of how systemic misalignment shapes admission outcomes and ongoing hospital pressures.

### 4.1 Limitations

This study has several limitations. First, the sample comprised RACH managers from a single region (Gold Coast, Queensland, Australia), which may limit generalisability to other contexts, particularly regions with different healthcare systems or aged care structures. Secondly, the interviewers were hospital‐based social workers with prior professional experience working with RACH managers. While this insider knowledge enabled contextually informed probing of operational and structural mechanisms, it may have influenced participants' responses. Reflexive practices were employed to minimise this risk, consistent with critical realist methodology. Thirdly, the data reflect participants' situated interpretations of the admission process rather than objective measurements of organisational practices. Critical realism recognises that these accounts provide valuable insights into underlying structural and organisational mechanisms but are necessarily filtered through individual perspectives and may not capture all aspects of systemic processes. Finally, although sample size was guided by information power, the relatively small number of participants may have limited the breadth of experiences captured.

Despite these limitations, the study provides a robust, contextually grounded understanding of the mechanisms shaping hospital‐to‐RACH admissions.

## 5 Conclusions

This study extends the evidence base on hospital‐to‐RACH transitions by repositioning the locus of analysis from hospital discharge processes to the mechanisms that shape managerial decision‐making. Using a critical realist approach, it demonstrates how hospital‐to‐RACH admission outcomes were produced by interacting structural and organisational forces mechanisms, operating across the macro (structural), meso (organisational), micro (relational) and interface (operational) levels. Managers in this study navigated admission decisions within constraints of bed availability, funding classifications, workforce capacity, regulatory governance, hospital discharge pressures and organisational directives.

Importantly, the study offers a multi‐level explanatory model that conceptualises the hospital‐to‐RACH admission pathway, highlighting the multifaceted considerations RACH managers must navigate. This model may assist policymakers and hospital administrators in understanding why RACH managers cannot always prioritise hospital‐referred patients over others already awaiting placement in the community, irrespective of pressures and priorities to reduce hospital wait times.

The implications for policy and practice are therefore layered. Structural reform is required to address funding misalignment, capacity constraints, and workforce limitations that narrow admission thresholds. Organisational supports that strengthen risk management capability and regulatory alignment may assist facilities to safely accommodate higher‐acuity residents. At the relational and interface levels, improved coordination, shared planning and clearer information exchange between hospitals and RACHs may reduce avoidable delays where placement capacity exists. Aligning hospital performance imperatives alongside aged care sector capabilities is essential to ensure equitable, timely and sustainable access to permanent residential care for older adults living in Australia.

## Funding

This project received clinical backfill funding from Gold Coast Health Allied Health Research.

## Disclosure

Use of AI: The authors confirm that no generative artificial intelligence tools were used in the writing or preparation of this manuscript.

## Ethics Statement

This study was approved by the Gold Coast Hospital and Health Service Human Research Ethics Committee on 28 August 2024, ((LNR) HREC/2024/QGC/109508).

## Conflicts of Interest

The authors declare no conflicts of interest.

## Supporting information


**Appendix S1:** Exploring patient discharge to RACH–interview questions.

**Appendix S2:** Consolidated criteria for reporting qualitative studies (COREQ): 32‐item checklist.

## Data Availability

The data that support the findings of this study are available on request from the corresponding author. The data are not publicly available due to privacy or ethical restrictions.
