# Supplementary material for: Beyond the Hospital Residential Aged Care Home Referral: A Critical Realist Exploration of Residential Aged Care Admission Practices Directly From Hospital
Source: Australas J Ageing. 2026 Jul 11;45(3):e70203. doi: 10.1111/ajag.70203 (PMC13355324; doi:10.1111/ajag.70203)
Supplement: Supplementary file 1 — Appendix S1: Exploring patient discharge to RACH–interview questions. [file AJAG-45-0-s002.docx]

**Exploring patient discharge to RACH –interview questions**

Please note, these are example questions/script and may be modified depending on interview responses.

**Introduction:**

- Thank you for your time today.
- My name is .......and I am a social worker at Gold Coast Health Service. I am also one of the investigators on this project. As you’re aware in participant information sheet that you have received, the purpose of this interview is to gain greater understanding of RACH managers perception and experience in accepting new residents directly following a discharge from Gold Coast Health Service hospital at either Gold Coast University Hospital (GCUH) or Robina Hospital (RH).
- There’s no right or wrong perspective, so feel free to share different viewpoints and be as open and honest as possible.
- The interview will be audio recorded; all data will be coded and will only be accessible by research team members. When we disseminate our findings, all data will be deidentified.
- This interview is completely voluntary so if any time you wish to stop feel free to do so, you do not need to provide a reason. Just let me know.
- Remind people of confidentiality
- I would also like to reassure you that your answers will be de-identified, analysed and themed and will not impact your relationship with GCHHS in what you may wish to share with me today.

Pre-Interview information to be collected:

1. Name:
2. Gender: ¨ Male ¨ Female ¨ Other
3. RACF Name:
4. RACF Business Group:
5. Are you responsible for RACF New Admission: ¨ Yes ¨ No
6. If yes, your role is: ¨ Administrative ¨ Clinical Assessment ¨ Both
7. How many years' experiences do you have in your current role:

¨ 0-1 ¨ 1-3 ¨ 4-6 ¨ 7-9 ¨ ≥ 10

1. Have you admitted residents directly from a GCHHS Hospital? If yes

¨ Gold Coast University Hospital

¨ Robina Hospital

1. What is your qualification?

**Hit record x 2:**

**I have hit the record button, and we are now recording is this Ok with you? Let's start!**

**Question:**

1. Could you describe the process for admitting a new resident to your facility?
   1. Prompt: what needs to be in place before you can accept a new resident?
   2. Which patients can you accept, for example, are there certain patients that you do not have the skill-mix to support such as patients with responsive behaviours, PEG Feed, Tracheostomy, Bariatric, etc.
2. How would you describe your experience accepting new resident referrals from the Gold Coast Health Service?
   1. Prompt: How do you manage hospital enquiries to your facility.
3. In what ways is a RACH admission enquiry from the hospital different from the community?
4. Prompt: what factors influence prioritising a potential new resident over others?
5. What do you think are some of the barriers to accepting new referrals from Gold Coast Health?
   1. What are some of factors that may make it harder for RACHs to accept new residents directly from hospital?
6. What may assist RACHs to accept new referrals from Gold Coast Health?
   1. Prompt- what types of documents/approvals in place make it easier to accept residents directly from hospital? Example - completion of Centrelink forms, providing Behaviour support plan, providing nursing care plan, ACAT codes, ACAT support plan etc.
7. Are there any things that you can suggest that might help support timely admissions of new residents directly from a Gold Coast Health Hospital to your facility
8. Prompt: can you suggest relevant areas for improvement for effective admissions of new residents from Gold Coast Health to your facility. Is there anything further you would like to add that we have not discussed that would make a difference to the current process of accepting new residents waiting for RACH placement directly from hospital?
9. In your opinion, what is the ideal process for admitting a new resident into your facility?

**Thank you for your time in participating in this research do you have any questions for me now that we have finished the formal part interview**
