# Supplementary material for: Beyond the Hospital Residential Aged Care Home Referral: A Critical Realist Exploration of Residential Aged Care Admission Practices Directly From Hospital
Source: Australas J Ageing. 2026 Jul 11;45(3):e70203. doi: 10.1111/ajag.70203 (PMC13355324; doi:10.1111/ajag.70203)
Supplement: Supplementary file 2 — Appendix S2: Consolidated criteria for reporting qualitative studies (COREQ): 32‐item checklist. [file AJAG-45-0-s001.docx]

Consolidated criteria for reporting qualitative studies (COREQ): 32-item checklist

**Developed from:**

Tong A, Sainsbury P, Craig J. Consolidated criteria for reporting qualitative research (COREQ): a 32-item checklist for interviews and focus groups. *International Journal for Quality in Health Care*. 2007. Volume 19, Number 6: pp. 349 – 357

**MANUSCRIPT TITLE: Beyond the Hospital Residential Aged Care Home Referral: A Critical Realist Exploration of Residential Aged Care Admission Practices Directly from Hospital**

| **No. Item** | **Guide questions/description** | **Reported on Page #** |
| --- | --- | --- |
| **Domain 1: Research team and reﬂexivity** |  |  |
| *Personal Characteristics* |  |  |
| 1. Inter viewer/facilitator | Which author/s conducted the interview or focus group? | p4  Natalia Mendez Arostica & Gunjan Patil |
| 2. Credentials | What were the researcher’s credentials? (E.g. PhD, MD) | p1 (title page)  **Angel Carrasco**  *BSW, MMH (Psychotherapy), Grad.Cert.(Dis,Mgt)*  ***Rachna Kumar***  **Natalia Mendez Arostica**  *BPsychSci, Grad Cert Adv HRM, MBA, MSW*  **H Laetitia Hattingh**  *BPharm, MPharm, GCertAppLaw, PhD*  ***Gunjan Patil***  *Diploma in Mechanical Engg, BA-majors in Sociology and Psychology, MSW*  ***Sarah Richards*** |
| 3. Occupation | What was their occupation at the time of the study? | p1 (title page)  **Angel Carrasco,** Director of Social Work Services**,**  **Dr Laetitia Hattingh, A/ Director of Allied Health Research**  **Rachna Kumar,** A/Social Worker Advanced Aged Care  **Natalia Mendez, Arostica** Social Worker Robina Hospital  **Gunjan Patil,** Social Worker Placement Support Team |
| 4. Gender | Was the researcher male or female? | 1 Male, 5 Females |
| 5. Experience and training | What experience or training did the researcher have? | p1,4  Research experience, in the context of education and career, gained through participation in studies, projects, or academic programs, and practical involvement in research activities. |
| *Relationship with participants* |  | p4  Professional relationship as external stakeholders in the local community. |
| 6. Relationship established | *Was a relationship established prior to study commencement?* | p4  In most cases, there was a professional relationship stablished prior to study commencement, between nursing home staff as care providers. |
| 7. Participant knowledge of the interviewer | *What did the participants know about the researcher? (e.g. personal goals, reasons for doing the research).* | p4  They knew the purpose of the study, reason for their inclusion, broader research goals and motives, nature of their participation, as well as the researcher transparency. |
| 8. Interviewer characteristics | *What characteristics were reported about the interviewer/facilitator? (e.g. Bias, assumptions, reasons and interests in the research topic)* | p4  Researchers informed participants about the credentials and roles, including that they were clinicians from Gold Coast Health, Qualified social workers, based in the Social Work Service at Gold Coast Hospital and Health Service, and that they have experience in RACF placement processes. |
| **Domain 2: Study design** |  |  |
| *Theoretical framework* |  |  |
| 9. Methodological orientation and Theory | *What methodological orientation was stated to underpin the study? (e.g. grounded theory, discourse analysis, ethnography, phenomenology, content analysis).* | p3  The study employed an interpretive qualitative approach informed by Critical Realist theory. Qualitative semi-structured interviews were conducted. The COREQ (*CO*nsolidated criteria for *RE*porting *Q*ualitative research) guidelines were utilised as the framework to underpin the research. Interviews were conducted face to face and online using Microsoft Teams by members of the research team. All interviews were recorded with participants’ consent and transcribed using Microsoft TEAMS and checked by the interviewing researchers for transcription accuracy. The interview questions and guide were designed prior to the interviews. Data were analysed inductively and thematically using the principles of reflexive thematic analysis. Two researchers conducted the initial coding and theme development, with a third researcher reviewing the coding to ensure consistency and analytical rigour. NVivo software was used to support data management and facilitate the thematic analysis. |

| *Participant selection* |  |  |
| --- | --- | --- |
| 10. Sampling | *How were participants selected? (e.g. purposive, convenience, consecutive, snowball)* | p4  The study was based on a purposive and convenience sample |
| 11. Method of approach | *How were participants approached? (e.g. face- to-face, telephone, mail, email)* | p4  email |
| 12. Sample size | *How many participants were in the study?* | p4  15 |
| 13. Non-participation | *How many people refused to participate or dropped out? Reasons?* | None |
| *Setting* |  |  |
| 14. Setting of data collection | *Where was the data collected? (e.g. home, clinic, workplace)* | p4  Workplace |
| 15. Presence of non- participants | *Was anyone else present besides the participants and researchers?* | No |
| 16. Description of sample | *What are the important characteristics of the sample? (e.g. demographic data, date)* | p4,5  The sample were 15 RACH managers on the Gold Coast, Queensland, Australia, playing a pivotal role in the timely acceptance of hospital-referred patients awaiting permanent placement.  The sample represented 28% of all RACHs in the region and was based on achieving qualitative data saturation. It included a wide variety of service types, such as not-for-profit and Commonwealth-funded providers, as well as standalone RACHs and those with co-located services such as independent living units. |
| *Data collection* |  |  |
| 17. Interview guide | *Were questions, prompts, guides provided by the authors? Was it pilot tested?* | p3  There was an interview guide provided by email prior to the interview. Both interviewers performed test pilots prior to the study commencement. |
| 18. Repeat interviews | *Were repeat interviews carried out? If yes, how many?* | No |
| 19. Audio/visual recording | *Did the research use audio or visual recording to collect the data?* | p4  All interviews were recorded with participants’ consent and transcribed using Microsoft TEAMS and checked by the interviewing researchers for transcription accuracy. |
| 20. Field notes | *Were ﬁeld notes made during and/or after the interview or focus group?* | p4  During and after |
| 21. Duration | *What was the duration of the inter views or focus group?* | p4  The in-depth, semi-structured interviews lasted between 35 and 90 minutes. |
| 22. Data saturation | Was data saturation discussed? | p4  Saturation was reached through ongoing thematic analysis during data collection, combined with deliberate sampling of diverse and knowledgeable participants, and validation via team discussions. |
| 23. Transcripts returned | *Were transcripts returned to participants for comment and/or correction?* | No |
| **Domain 3: analysis and ﬁndings** |  |  |
| *Data analysis* |  |  |
| 24. Number of data coders | How many data coders coded the data? | P4  Two researchers conducted the initial coding and theme development, with a third researcher reviewing the coding to ensure consistency and analytical rigour. |

| 25. Description of the coding tree | *Did authors provide a description of the coding tree?* | No |
| --- | --- | --- |
| 26. Derivation of themes | *Were themes identiﬁed in advance or derived from the data?* | p4,5  In advance and modified from the data |
| 27. Software | *What software, if applicable, was used to manage the data?* | p4  NVivo software was used to support data management and facilitate the thematic analysis. |
| 28. Participant checking | *Did participants provide feedback on the ﬁndings?* | No |
| *Reporting* |  |  |
| 29. Quotations presented | *Were participant quotations presented to illustrate the themes/ﬁndings? Was each*  *quotation identiﬁed? (e.g. participant number)* | Pp-9  Yes |
| 30. Data and ﬁndings consistent | *Was there consistency between the data presented and the ﬁndings?* | p6-9  Yes |
| 31. Clarity of major themes | *Were major themes clearly presented in the ﬁndings?* | p6-9  Yes |
| 32. Clarity of minor themes | Is there a description of diverse cases or discussion of minor themes? | p6-9  No |
